# The Influence of Proteolytic Malt Modification on the Aging Potential of Final Wort

**DOI:** 10.3390/foods10102320

**Published:** 2021-09-29

**Authors:** Arndt Nobis, Florian Lehnhardt, Marcel Gebauer, Thomas Becker, Martina Gastl

**Affiliations:** Brewing and Beverage Technology, TU Munich, Weihenstephaner Steig 20, 85354 Freising, Germany; arndt.nobis@tum.de (A.N.); florian.lehnhardt@tum.de (F.L.); gebauer-marcel@web.de (M.G.); tb@tum.de (T.B.)

**Keywords:** beer aging, wort boiling, Maillard reaction, brewing, dicarbonyls, bound-state aldehydes, malt modification

## Abstract

The dynamic changes in beer flavor are determined by its aging potential, which comprises of present free and bound-state aldehydes and their precursors. Rising flavor-active aging compounds cause sensory deterioration (flavor instability). These compounds are mainly formed upstream in the brewing process through the Maillard reaction, the Strecker degradation, or lipid oxidation. Wort boiling is an especially critical production step for important reactions due to its high temperature and favorable pH value. Amino acid concentration, as an important aging-relevant precursor, is variable at the beginning of wort boiling, mainly caused by the malt modification level, and can further influence the aging potential aging formation during wort boiling. This study investigated the effect of the proteolytic malt modification level on the formation of precursors (amino acids and dicarbonyls) and free and bound-state aldehydes during wort boiling. Six worts (malt of two malting barley varieties at three proteolytic malt modification levels) were produced. Regarding precursors, especially Strecker, relevant amino acids and dicarbonyls increased significantly with an enhanced malt modification level. Concentrations of free and bound aldehydes were highest at the beginning of boiling and decreased toward the end. A dependency of malt modification level and the degree of free and bound aldehydes was observed for 2-methylpropanal, 2-methylbutanal, and 3-methylbutanal. Generally, a higher proteolytic malt modification level tended to increase free and bound aldehyde content at the end of wort boiling. Conclusively, the aging potential formation during boiling was increased by an intensified malt modification level.

## 1. Introduction

**Figure 1:**
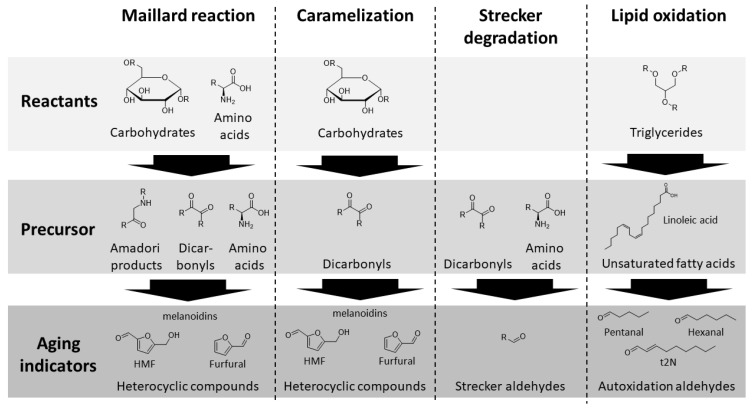
Overview of selected aging-relevant reactions and their key reactants, precursors, and aging indicators.

During storage (after bottling), beer flavor undergoes dynamics, including concentration decrease or increase in various flavor-active substances [1]. This flavor instability causes sensory beer deterioration and occurs mostly due to rising aldehydes, defined as aging indicators [2]. Longer distribution distances and periods intensified the problem for brewing industries. Figure 1 shows an overview of reactants, precursors, and aging indicators of four selected key aging reactions, the Maillard reaction, caramelization, the Strecker degradation, and lipid oxidation, which contribute to aldehyde formation during beer aging [3,4].

Alongside the final stage of aging indicators, the concentration of precursors, such as dicarbonyls or amino acids, play a key role in flavor instability during beer storage [1]. In the former, for precursor formation, the level of reactants, such as carbohydrates and amino acids, is important, whereby the substance class of amino acids can act in both functionalities (reactant and precursor). The important reactions (Figure 1) are subsequently described in further detail.

The oxidation of unsaturated fatty acids occurs enzymatically [5] or by autoxidation [2]. Important precursor compounds include linoleic acid and linolenic acid, which mainly originate from malt. Their oxidation forms aroma-active aging aldehydes, such as (E)-2-nonenal (t2N), pentanal (Pent), hexanal (Hex), and heptanal (Hept) [6,7]. During the Maillard reaction, the nucleophilic amino group of amino acids, as reactants, can react with the carbonyl group of carbohydrates to form Amadori products [8,9]. These compounds can be further degraded to dicarbonyl compounds. The dicarbonyls are a various substance class, with important representatives being 3-desoxypentosone (3-DP), 3-deoxyglucosone (3-DG), or 1-deoxyglucosone (1-DG). Regarding occurring concentrations, 3-DG is the predominant dicarbonyl in beer and wort [10,11,12]. These dicarbonyls are important precursor compounds [13] and are already formed during malting [14]. The dicarbonyls can also occur directly by carbohydrate dehydration during caramelization [8]. They can further react in several pathways, such as dehydration reactions [15] or the Strecker degradation [16]. In contrast, C5-dicarbonyls mainly result in furfural (Fur), while C6-dicarbonyls produce 5-hydroxymethylfurfural [15] by dicarbonyl dehydration. Regarding the Strecker degradation, amino acids, as precursors, are degraded to Strecker aldehydes. Typical aroma-active compounds are 2-methylpropanal (2MP) from valine, 2-methylbutanal (2MB) from isoleucine, 3-methylbutanal (3MB) from leucine, methional (Meth) from methionine, and phenylacetaldehyde (PA) from phenylalanine [17].

Regarding all reactions, the reactivity of reactants requires high activation energies [3]. Therefore, forming precursors and aldehydes upstream is easier during beer production due to its higher thermal processing when compared to beer storage. Wort boiling, especially, provides the best conditions for forming aging aldehydes and their corresponding precursors due to its intensive thermal load and favorable pH level of 5.4–5.8 for the described aging-relevant reactions, such as dehydration. Alongside its elevated reaction potential, high concentrations of reactants (low molecular carbohydrates and amino acids) at the beginning of boiling induce the best conditions for precursor and aging aldehyde formation [2,9,18]. Thus, aging aldehydes are already formed during wort boiling in high concentrations [19]. These high concentrations in the final wort could be critical for flavor instability; however, it should be considered that yeast partly reduces the aldehydes afterward in the early stage of fermentation [20]. Despite the reduced activity of yeast, the formed aldehyde concentration is important because they can be present in a bound-state due to chemical equilibrium. Here they overcome fermentation in these forms and get re-released during beer aging [2]. Possible reactions include forming cysteinylated aldehydes [21], bisulfite adducts [22], and imine formation [23]. Suda et al. showed that 85% of the wort aldehydes found in wort were transferred to beer by their bound-state form [24]. Furthermore, Baert et al. pointed out that imine and bisulfite adducts were the most important masking reactions in beer and wort [2]. In particular, imine formation of aldehydes could be important in wort because of the high concentrations of free amino acids [18]. Disadvantageously, imine adducts are more stable at high pH values (pH > 7) [25]. Regarding bisulfite formation, studies showed that sulfur dioxide (SO_2_) formation by sulfate reduction in yeast cells occurs mainly in an intermediate stage in the fermentation after yeast cell growth ceases [26,27]. Compared with the imines, the bisulfite adducts show a constant equilibrium at a pH range of 4–6, which is more suitable for wort and beer [28]. Released bisulfite from yeast cells could immediately mask remaining aldehydes, whose reduction rates depend on the strain type, aldehyde type, and fermentation temperature [29]. Alongside the masking effects, the concentration of the formed free aldehydes during boiling could be used as a good indicator for evaluating flavor instability [30].

The formed precursors, free aldehydes, and bound-state aldehydes influence the flavor instability in beer. Therefore, this study defines the sum of the concentration of the three classes as the aging potential. The aging potential is a dynamic result of the brewing process; the ratios of the three classes, and their absolute concentration, changes during the brewing process and aging. Until now, previous studies focused on the investigation of different boiling systems and thermal load [22,31,32], single-substance model-boiling experiments to verify reaction pathways [33,34], or the influence of oxygen [35] on aging potential formation during boiling. However, the influence of a varied reactant concentration, such as amino acid content, was not investigated. Here, the malt modification level by varying the steeping degree is prone to be a suitable parameter because a higher level causes increased nitrogen [36] and peptide contents in malt [37]. This nitrogen potential will be further enzymatically degraded to amino acids during mashing and result in a higher concentration of this substance class at the beginning of wort boiling.

This study hypothesized that a higher content of amino acids, as reactants, directly influences the final wort’s aging potential by an increased malt modification level. Thus, the study investigated the influence of a varied reactant concentration on the aging potential formation during wort boiling.

## 2. Materials and Methods

### 2.1. Chemicals

All amino acids (L-form), [13C, 15N], labeled amino acids, D-glucose, potassium dihydrogen phosphate, methanol (liquid chromatography–mass spectrometry (LC-MS) grade), o-phenylenediamine (OPD), furfural, pentanal, hexanal, heptanal, (E)-2-nonenal, hydrochloric acid, 4-fluorobenzaldehyde, 2-isobutyl-1,3-thiazolidine-4-carboxylic acid (3MB-CYS; 95%), 2-(2-(methylthio)ethyl)-1,3-thiazolidine-4-carboxylic acid (MET-CYS; 95%), 2-pentyl-1,3-thiazolidine-4-carboxylic acid (HEX-CYS; 95%), caffeine, ethanol (absolute), disodium hydrogen phosphate dihydrate, acetic acid, 2-methylbutanal (2MB), 3-methylbutanal (3MB), 2-methylpropanal (2MP), phenylacetaldehyde (PA), and water (LC-MS grade) were obtained from Merck (Darmstadt, Germany). Acetonitrile used for LC-MS analysis was purchased from VWR (Darmstadt, Germany). Additionally, 2-phenyl-1,3-thiazolidine-4-carboxylic acid (BEN-CYS; 97%), 2-benzyl-1,3-thiazolidine-4-carboxylic acid (PHE-CYS; 97%), and 2-(2-furanyl)-1,3-thiazolidine-4-carboxylic acid (FUR-CYS; 95%) were purchased from Th.Geyer (Berlin, Germany), while the 2-isopropyl-1,3-thiazolidine-4-carboxylic acid (2MP-CYS; 95%) was purchased from ABCR (Karlsruhe, Germany). The 3-DG (>95%) was obtained from Apollo Scientific Ltd. (Cheshire, UK), and the 3-DGal (3-deoxygalactosone; >90%) was purchased from Carbosynth Ltd. (Berkshire, UK). Before use, the water for analytics was purified using a micropore water purification system (Thermo Fisher Scientific Inc., Waltham, MA, USA).

### 2.2. Malt Production

Six malts with different proteolytic modification levels were produced in a pilot-malting system. The malts comprised of two malting barley varieties (harvest year 2019): B1 (Avalon, Nordsaat Saatzucht GmbH, Langenstein, Germany) and B2 (Marthe, Saatzucht Josef Breun GmbH and Co. KG, Herzogenaurach, Germany) with different genetic modification characteristics (high and medium modification levels). The barley varieties had the same protein content of 10% d.m. Both were further targeted and modified, reaching different proteolytic modification levels (P1: low, P2: medium, and P3: high) by varying the steeping degree (Table 1). These variations changed the concentration of reactants (amino acids) and reactant formation potential by protein solubilization (proteolytic enzyme activity) during mashing. All malt samples were malted as standard, according to MEBAK R-110.00.008 (016–03), and standard malt parameters were analyzed on the basis of the isothermal 65 °C laboratory mashing regime R-207.00.002 (2016-03) analogous to common variety evaluation in barley-breeding programs [38]. B1 was germinated at steeping degrees of 38% (P1), 41% (P2), and 44% (P3), while B2 was germinated at steeping degrees of 39% (P1), 43% (P2), and 47% (P3) to obtain the target values of the soluble nitrogen content. Table 1 summarizes the soluble nitrogen targets and the reached values of the malt variations (B1P1, B1P2, B1P3, B2P1, B2P2, and B2P3) in the study.

The required specification of soluble nitrogen for brewing purposes of barley malts is 580–680 mg/100 g dry weight (according to isothermal 65 °C mashing procedure) [39,40]. Compared with the target values, the P3 variations of B1 were slightly decreased, and the P3 variation of B2 was slightly increased, but they differed significantly from the P2 samples.

### 2.3. Wort Production and Sampling

Ten kilograms of produced malts were milled using a type 16/16 two-roller mill from Künzel (Kulmbach, Bayern). The pilot brewhouse (80 L) was used as previously described in [13]. The grist was mashed in with 40 L standardized brewing liquor at 60 °C, and the temperature was raised to 62 °C. Two rests of 30 min each were held at 62 °C and 72 °C, after which the mash was raised to 78 °C and held for 10 min. The heating rate between rests in the mash tun was set to 1.7 °C/min. The mash was then transferred to the lauter-tun, preheated to 78 °C, and a lauter rest of 10 min was held. Lautering was performed with two sparges of 15 L and a third sparge of 14 L with brewing liquor at 78 °C until a target extract content of 10.5° P was reached. A rake was used during the third sparge, and, while collecting the last sparge, the kettle was heated to near-boiling temperatures (95 °C) and brought to a rolling boil after the lautering was finished. Taurus (13.0% α-acids; 33.3 g) was added at the beginning of the 60 min boiling time to reach 15 international bitter units (IBU). The evaporation rate was 10.6% during the 60 min of boiling. All brewhouse steps were standardized for variations in this study. Sampling was done every 10 min up to the end of boiling in a standardized procedure. The sampling containers were 50 and 2 mL plastic tubes used for each of the three sampling times. Sampling was done using a self-manufactured sampling stick. The samples were filtered using a folded filter paper and immediately frozen after filtration. The brewing trials were done in duplicates.

### 2.4. Quantitation of Free Aldehydes by HS-SPME-GC-MS

The procedure was performed according to Lehnhardt et al. [4], with minor changes. The cooled wort sample (5 mL) was transferred with 50 µL internal standard (2 mg/L p-fluorobenzaldehyde in ethanol) to a 20 mL headspace vial and stored in a cooled autosampler tray (17 °C). Extraction was performed using a CAR-PDMS-DVB fiber. First, the fiber was loaded with o-(2,3,4,5,6-pentafluorobenzyl)hydroxylamine (PFBHA) for 10 min at 40 °C. Afterward, the headspace of the sample was extracted for 30 min at 40 °C. Next, the fiber was injected with a 1/5-split at 270 °C into a GC (GC-Ultra 1300, Thermo Scientific Inc., Waltham, MA, USA) coupled to a single quad mass spectrometer (ISQ 7000, Thermo Scientific Inc., Waltham, MA, USA). The GC was equipped with a DB-5 column (length, 60 m; inner diameter, 0.25 mm; and film thickness, 0.25 µm; Thermo Scientific Inc., Waltham, MA, USA). The carrier gas used was helium (flow rate 1.85 mL/min). The starting temperature was held at 60 °C for 4 min, followed by heating at 5 K/min to a final temperature of 250 °C, which was maintained for 3 min. A full scan mode (m/z 35–350), with a dwell time of 0.02 s, was applied to the analysis. Each sample was analyzed in triplicate. Peak detection was performed in Xcalibur 3.1.66.10 (Thermo Scientific Inc., Waltham, MA, USA).

### 2.5. Quantitation of Bound-State Aldehydes after Release with 4-Vinylpyridine (4-VP)

The procedure was performed as described in the previous section, with one exception. Before adding the internal standard, a 4-VP solution was added (50 µL, 1/1 4-VP/ethanol, *v*/*v*). These samples were incubated in the autosampler tray at 17 °C for at least 6 h before analysis. During the elution of 4-VP from the GC column, mass spectrometric detection was turned off at 13 to 14 min. The concentration of bound-state aldehydes was the difference between the aldehyde contents after 4-VP release and the content of its free form.

### 2.6. Quantitation of 3-DG and 3-DGal

High-performance liquid chromatography, with ultraviolet detection (HPLC-UV) analytics and sample preparation, were applied as previously performed by Degen, Hellwig, and Henle [11] and modified as published by Nobis et al. [13]. Wort samples were measured directly after derivatization and filtration (0.45 µm).

### 2.7. Quantitation of Amino Acids

Nineteen amino acids were determined using HPLC-tandem mass spectrometry (MS/MS) in the multiple reaction monitoring mode as previously published by Nobis et al. [14].

### 2.8. Quantitation of Cysteinylated Aldehydes

UPLC-Q-ToF analysis of cysteinylated aldehydes was performed on a Waters Acquity UHPLC-H system coupled to a Xevo G2-XS Q-TOF (Waters Corporation, Manchester, UK). An Acquity BEH C18 column (2.1 mm × 150 mm, 1.7 μm) was used for chromatographic separation. The samples were kept at 10 °C, and the injection volume was 1 µL. Mobile phase (A) water + 0.1% *v*/*v* formic acid and (B) acetonitrile + 0.1% *v*/*v* formic acid were used with the following gradient: 0–1 min 90% A + 10% B, 1–6 min linear gradient to 48% A + 52% B, 6–6.5 min linear gradient to 100% B, 6.5–7.5 min 100% B, 7.5–7.6 min linear gradient to 90% A + 10% B, and 7.6–8.5 min 90% A + 10% B. The flow rate was kept constant at 0.4 µL/min, and the column temperature was set to 40 °C. Analytes were ionized using an electrospray ionizer in positive mode. Capillary and sample cone voltages were 1 kV and 40 V, respectively. The ion source was kept at 120 °C, and the desolvation temperature was 450 °C. The cone and desolvation gas flow (N2) were 50 and 500 L/h, respectively. The mass range was m/z 50–1200 with a scan time of 0.15 s using resolution mode.

The molecular structures of the seven cysteinylated aldehydes and caffeine, and the m/z of the molecular ion [M + H]^+^, were calculated. The retention times and m/z of the respective molecular ions are shown in Table 2. Additionally, sample and calibration data were searched for respective m/z, and the response values were used for screening analysis or quantification.

The calibration was done by preparing a stock solution of the seven cysteinylated aldehydes: 2MP-CYS, MET-CYS, FUR-CYS, 3MB-CYS, BEN-CYS, PHE-CYS, and HEX-CYS, with a final concentration of 100 µg/L in LC-MS-grade water with 5% ethanol. This stock solution was then diluted to obtain four calibration points with concentrations of 0.5, 1, 5, and 10 µg/L of each cysteinylated aldehyde. Next, caffeine, which was used as an internal standard, was prepared in LC-MS-grade water with 5% ethanol and a final concentration of 100 µg/L. For calibration, the internal standard was added to each cysteinylated aldehyde mixture at a concentration of 5 µg/L.

The samples were filtered through a polyamide filter (0.2 µm) and then spiked with the internal standard solution to achieve a caffeine concentration of 5 µg/L. Then, samples were thoroughly mixed and used directly for UPLC-Q-ToF analysis.

### 2.9. Influence of Varied Reactants during Wort Boiling on Aging Potential

Verifying the dynamic changes in the aging potential during wort boiling was performed by boiling experiments on a laboratory scale with spiked reactants and precursor compounds. The B2P3 malt was milled using a DLFU disk mill from Bühler (Braunschweig, Germany) at a disk gap of 0.2 mm. Fifty grams of grist was mashed with 200 mL tempered distilled water in a laboratory mash procedure (30 min, 62 °C; 6 min, 63 °C–72 °C; 30 min, 72 °C; 5 min, 72 °C–78 °C; and 2 min, 78 °C). The produced mash was filtered using a laboratory filter (folded paper filter) from VWR International GmbH (Darmstadt, Germany) and diluted with water to a target extract content of 12.5° P. Several reactants and precursor compounds were spiked by increasing the initial concentration by 100%, 200%, and 400%. The reactants and precursors used were valine (2-MP), isoleucine (2-MB), leucine (3-MB), methionine (methional), phenylalanine (PA), lysine (3-DG, 3-DGal, and Strecker aldehydes), 3-DG (Strecker aldehydes), fructose (3-DG, 3-DGal, and Strecker aldehydes), glucose (3-DG, 3-DGal, and Strecker aldehydes), arabinose (furfural and Strecker aldehydes), and linoleic acid (lipid oxidation aldehydes). The initial concentrations of amino acids and dicarbonyls were determined as previously described. Sugars (glucose: 9 g/L; fructose: 2 g/L; arabinose: 60 mg/L) and linoleic acid (1 mg/L) were defined according to the literature [18]. All variations were boiled in sealed tubes in triplicate and analyzed after 60 min of boiling. Aging aldehydes and dicarbonyls were determined as described in the previous sections.

### 2.10. Statistical Analysis

Statistical analysis was performed using JMP Pro v.14 (SAS Institute GmbH, Heidelberg, Germany). Results were presented as average ± standard deviation. ANOVA (Tukey test), at a significance level of 0.05, was used for average comparisons.

## 3. Results and Discussion

Upon beer aging, aldehyde levels in beer increased and caused undesired flavor changes. Wort boiling plays a major role in flavor instability of beer, as essential aging aldehyde precursors, such as amino acids and aging aldehydes, might be formed. The sum of the investigated precursor amino acids and dicarbonyls and free and bound-state aldehydes, formed during wort boiling, contributed to the aging potential for beer originating from wort.

### 3.1. Amino Acids in Wort Boiling Process

In industrial brewing, wort amino acid concentrations are influenced by multiple factors, such as raw material choice, grist load, and malt modification. Amino acids can contribute to aging potential either as reactants in the Maillard reaction or directly as precursors in the Strecker degradation. Therefore, this study monitored them during wort boiling due to different malt modification levels (P1, P2, and P3). Supplementary Materials showed all amino acid concentrations during wort boiling at all malt variations in this study. The concentration of almost all amino acids remained constant during wort boiling. Associating the evaporation rate during boiling, the amino acids were mainly degraded during wort boiling. This behavior indicates their reactivity in aging-relevant reactions, such as the Strecker degradation [41] or the Maillard reaction [8]. Alternatively, the amino acids react in oxidative pathways [8] or form imine adducts with carbonyls [23]. Glutamine, as an exception, was degraded more strongly than the other amino acids. This amino acid showed a linear degradation during wort boiling at all variations (B1P1, B1P2, B1P3, B2P1, B2P2, and B2P3). Presumably, the enhanced reduction was caused by the thermal catalyzed reaction of pyrrolidonecarboxylic acid. The amid function of glutamine, especially, is prone to undergoing internal cyclization, forming the pyrrolidone function [42,43].

Alongside the behavior during wort boiling, this study focused on investigating the influence of the proteolytic malt modification level on the amino acid concentration. Figure 1 shows the content of selected Strecker active amino acids (valine, isoleucine, leucine, phenylalanine, and methionine) at the beginning (0 min) and end (60 min) of wort boiling for different malt modification levels (P1 < P2 < P3). The content of the selected amino acids provides a higher aging potential because they can form Strecker aldehydes in the final product beer [44] and, therefore, influence flavor instability.

According to the described behavior during wort boiling, the concentrations at the beginning and end of boiling were comparable. Despite leucine at B1, the Strecker active amino acids showed increased concentrations by an enhanced malt modification level. In particular, the highest malt modification level (P3) demonstrated increased levels of the presented amino acids (Figure 1). The observed effects could be explained in two ways. First, an increased proteolytic malt modification already caused higher amino acid levels [14]. Suppose the possible formation or degradation reactions during mashing were equal because of standardized mashing procedures for all malt variations; in that case, the ratio of the single amino acid contents between the varied proteolytic malt levels remains constant during wort boiling. Thus, the described effect results in higher amino acid concentrations at the beginning and end of boiling caused by malts with higher proteolytic malt modification. Second, the accelerated protease activity of malts with higher malt modification levels [45] causes higher amino acid contents during mashing. Presumably, the increased contents of amino acids at the P3 variation were a combined effect of the two described ways. Investigating a calculatable influence of the proteolytic malt modification on amino acid concentration in the final wort, the soluble nitrogen content (Table 1), as a representative parameter for the malt modification level, was correlated with the concentrations of amino acids in the final wort at all variations. Table 3 shows the resulting correlation coefficients.

A high correlation coefficient at the end of wort boiling indicated that the reactivity of the amino acids during wort boiling was mainly affected by the proteolytic malt modification level. The Strecker active amino acids, valine, isoleucine, leucine, and phenylalanine, showed high correlation factors (Table 3). This result indicated that the intensity of the Strecker reaction was affected by the proteolytic malt modification level. Surprisingly, methionine, as another well-known Strecker active amino acid, showed a different behavior. Presumably, here, alternative reactions were advantaged, such as the oxidation of the sulfur atom to the sulfone group [46]. Regarding other amino acids, it can be assumed that they react more in non-proteolytic modification-influenced pathways, such as oxidation, esterification, or cleavage reactions. It could be indicated that proteolytic malt modification influenced the Strecker reactivity because it strongly influences the chemical reaction pathways of Strecker active amino acids.

### 3.2. Dicarbonyl Formation during Wort Boiling

**Figure 2:**
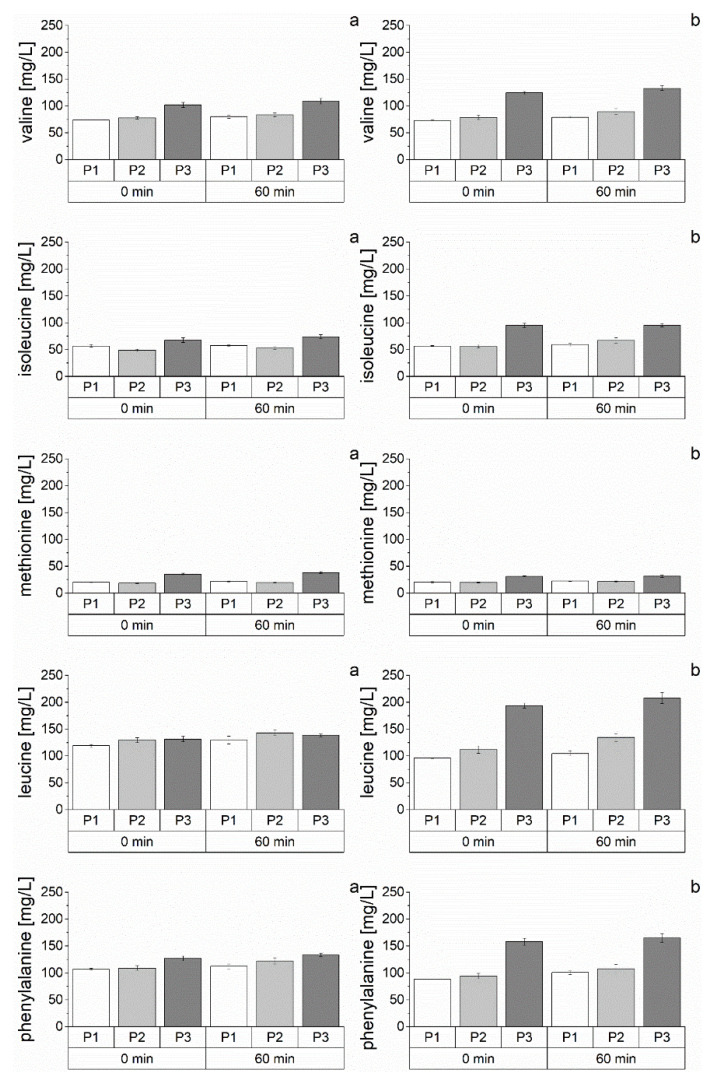
Concentration of selected amino acids at the start (0 min) and end (60 min) of boiling at different malt modification levels (P1 < P2 < P3; *n* = 3); (**a**) = B1; (**b**) = B2.

Aside from the amino acids, another important group of precursors is the dicarbonyl compounds. They play a key role in aging aldehyde formation during the Strecker degradation and the Maillard reaction. Thus, they directly contribute to aging potential because of their precursor activity. Therefore, the main dicarbonyls, 3-DG and 3-DGal, were monitored during wort boiling due to different malt modification levels (P1 < P2 < P3). Figure 2 shows the concentrations of 3-DG and 3-DGal during wort boiling at all proteolytic variations.

Comparing both compounds, it could be confirmed that 3-DG was the major dicarbonyl [10] because it occurred in higher concentrations during wort boiling. The 3-DGal was formed from 3-DG by interconversion [47] and, therefore, resulted in lower concentrations during wort boiling.

The 3-DG concentration showed a linear increase during wort boiling at all variations. The linearity combined its formation in the Maillard reaction or caramelization and the occurring evaporation effect. The formation of 3-DG indicated that the Maillard reaction was already at its advanced phase during wort boiling [8]. The higher proteolytic malt modification level showed a strong accelerating effect on the initial and final concentrations of the dicarbonyl. Presumably, the enhanced content of amino acids promoted its formation through the Maillard reaction. Additionally, 3-DG precursors, such as Amadori products, are increased by a higher malt modification level in malt [14] and can lead to higher 3-DG concentrations during wort boiling. The 3-DG formation rate was also increased by higher malt modification levels (B1P1: 0.09 mg/(min*L), B1P2: 0.10 mg/(min*L), B1P3: 0.17 mg/(min*L), B2P1: 0.10 mg/(min*L), B2P2: 0.12 mg/(min*L), and B2P3: 0.17 mg/(min*L)). This effect indicates that a higher proteolytic malt modification level forms an increased aging potential for the final beer due to 3-DG during wort boiling. Regarding the calculatable influence (correlation coefficient) of the malt modification level, the initial 3-DG concentration (R^2^ = 0.95), final 3-DG concentration (R^2^ = 0.97), and 3-DG formation rate (R^2^ = 0.81) showed good correlations with the soluble nitrogen content of the malts used (Table 1). Therefore, it could be concluded that the proteolytic malt modification level strongly influenced the Maillard and caramelization reactivity toward 3-DG formation during wort boiling in this study.

The second observed dicarbonyl 3-DGal was linearly formed during wort boiling, much like 3-DG. The occurring formation was a combined effect of the 3-DG interconversion and evaporation during boiling. The 3-DGal formation indicated that 3-DG formation and degradation took place simultaneously during wort boiling. The proteolytic P3 level showed the highest initial and final concentrations of 3-DGal. P1 and P2 showed no significant difference in their 3-DGal contents. It could be assumed that a certain level of 3-DG reactivity was needed to promote its interconversion to 3-DGal. The formation rates showed no differences and were calculated to an average of 0.04 mg/(min*L). The 3-DG formation was stronger than the 3-DGal formation, and the effect confirmed the importance of 3-DG as the major dicarbonyl during wort boiling. This study identified that a higher proteolytic modification level enhanced the aging potential toward the 3-DG and 3-DGal formation.

### 3.3. Formation of Free and 4-VP-Releasable Aging Aldehydes during Wort Boiling

The observed amino acids and dicarbonyls can react as precursors to aging indicators, such as aging aldehydes, which occur during wort boiling in free or bound-state forms. Bound-state aldehydes are releasable by adding 4-VP. After the release, they are detectable as free forms. Therefore, this study investigated the formation of free, 4-VP-releasable, and cysteinylated aging aldehydes during wort boiling. A high concentration of free and bound-state aldehydes contributes to the aging potential because of their functionality as free and releasable aging indicators. Table 4 shows the concentration of aging aldehydes of B1 and B2 at 0, 30, and 60 min of wort boiling. All concentrations during boiling are summarized in the Supplementary Materials section. The analytes comprised of Strecker aldehydes (2MP, 2MB, 3MB, methional, and PA), lipid oxidation aldehydes (t2N, pentanal, hexanal, and heptanal), and aldehydes derived from the Maillard reaction (furfural).

The concentration of all detected aldehydes at all variations (P1, P2, and P3) had a maximum concentration at the beginning of wort boiling and underwent a reduction until approximately 30 min and increased again until the end of boiling. The first decrease was caused by the evaporation effect of the boiling system. In particular, the vacuum pump usage of the pilot brewing system forced evaporation of the highly volatile aldehydes. However, it could be expected that formation reactions took place simultaneously in the first 30 min because of the previously described degradation effects of the amino acids. The first strong decreasing effect was observed in open boiling systems by de Schutter et al. [19]. After 30 min, the formation became more important than degradation or evaporation. The following increase could be caused by ongoing reactions: Strecker degradation, lipid oxidation, Maillard reaction, and caramelization. Alongside 2MP, the other aldehydes showed comparable levels with the literature values [19]. At the P3 level especially, 2MP occurred in strongly increased concentrations in our study.

The aldehydes derived from different reaction types will be discussed separately with regard to the proteolytic malt modification effect during wort boiling. Evaluating the Strecker aldehyde concentrations, the low (P1) and medium (P2) malt modification levels showed no significant difference for B1. However, B2 showed a significant increase in Strecker aldehyde concentration according to an enhanced proteolytic modification order (P1 < P2 < P3). Here, it should be considered that the difference in soluble nitrogen contents of the malts between the P1 and P2 levels, at variety B1, was lower than that at variety B2 (Table 1). Presumably, the difference in soluble nitrogen malt content for B1P1 and B1P2 was too low for differentiation within the Strecker aldehyde concentrations during wort boiling. However, the Strecker aldehydes were strongly increased in P3 (high modification) at both barley varieties. However, the strongly increased contents at the P3 level could be caused by the observed enhanced concentrations of amino acids and dicarbonyls as precursors.

The increased precursor pool accelerated the Strecker degradation during wort boiling. Furthermore, regarding the correlation with the soluble nitrogen content (Table 1) of the Strecker aldehyde concentration at the end of boiling, 2MP (R^2^ = 0.77), 2MB (R^2^ = 0.89), methional (R^2^ = 0.90), and PA (R^2^ = 0.87) showed a good correlation. Therefore, their formation was mainly influenced by the proteolytic malt modification level. Regarding the Maillard reaction, the highest contents for furfural were observed at P3 variations in this study at both barley varieties. The correlation coefficient of the furfural content to the soluble nitrogen content of the used malts (Table 1) was 0.70. This indicated that an increased proteolytic malt modification level accelerated the Maillard reaction during wort boiling due to the enhanced contents of amino acids and dicarbonyls as precursors. Lipid-oxidized aldehydes showed no consistent influence on malt modification. Contrary to the Strecker aldehydes, the highest values were observed at P1 for B1. Surprisingly, B2 showed an inverse effect with the highest levels at P3 variation. Presumably, the formation of lipid-oxidized aldehydes was independent of the malt modification level and was influenced more by barley variety or environmental growing conditions. An increased proteolytic malt modification level enhanced the aging potential originating from final wort toward free aldehyde formation during wort boiling by the Strecker degradation and the Maillard reaction.

**Figure 3:**
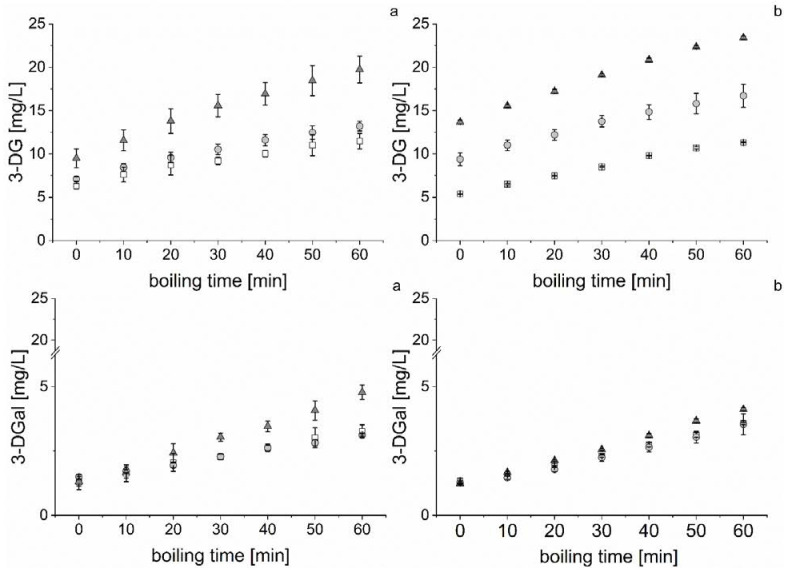
Formation of 3-DG and 3-DGal ((**a**) = B1, (**b**) = B2)) during wort boiling; squares: P1, points: P2, triangles: P3 (*n* = 3).

**Figure 4:**
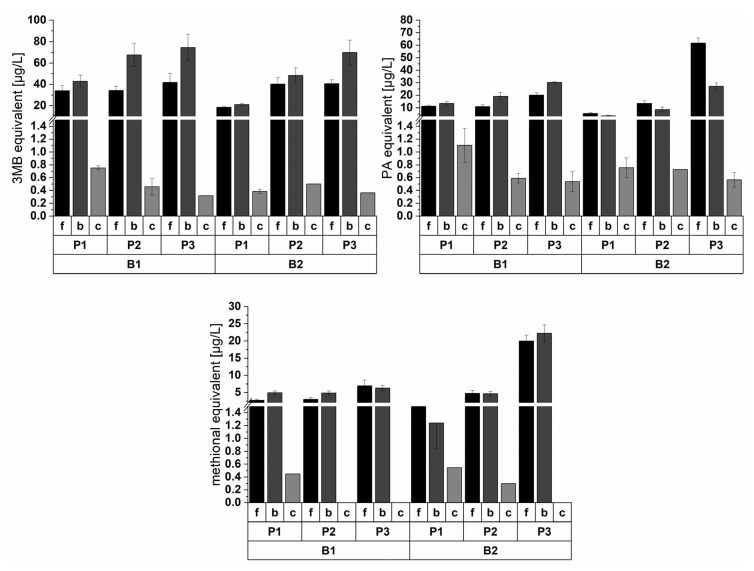
Concentration equivalent of free (f), 4-VP-releasable (b), and cysteinylated (c) forms of 3MB, PA, and methional at the end of boiling (*n* = 3).

Aside from their free form, the formed aldehydes during wort boiling can also be present in bound-state, such as imines or cysteinylated aldehydes [2]. These masked forms also directly contribute to the aging potential. Therefore, this study further investigated the bound-state form of aging aldehydes by release through 4-VP addition during wort boiling due to different malt modification levels (P1, P2, and P3). Figure 3 and Figure 4 show the concentrations of free, 4-VP-releasable, and cysteinylated forms of 3MB, PA, and methional at the end of boiling. Only the cysteine adducts occurred in quantifiable concentrations. The cysteinylated bound aldehyde was the calculated molar equivalent concentration from the determined cysteine adduct. All concentrations of 4-VP-releasable aldehydes are presented in the Supplementary Materials.

Regarding the 4-VP-releasable aldehydes, Strecker aldehydes and furfural concentrations were increased at the end of boiling by an enhanced malt modification level within a single barley variety. This could be caused by an enhanced imine formation due to higher amino acid contents. A correlation with the soluble nitrogen content of the used malt over both barley varieties could only be observed for 2MB (R^2^ = 0.91), methional (R^2^ = 0.82), and furfural (R^2^ = 0.85). Here, the formation of 4-VP-releasable aldehydes was affected by proteolytic malt modification. Lipid oxidation aldehydes showed no trend in their 4-VP-releasable form due to the malt modification. The ratio of free and 4-VP-releasable forms ranged broadly between 0.19 and 3.66, with an average of 0.95 for all aldehydes at all variations. Furfural and PA showed the highest ratios within the observed aldehydes. The range had a random distribution with no effect on the barley variety or proteolytic malt modification. The formation of imines was acidic-catalyzed and was favored at a pH value of 4–5 (an increase in electrophilicity of C-atom). However, the stability of the imine was increased at higher pH values up to 10 [2,25]. Presumably, the aldehydes underwent a dynamic equilibrium during wort boiling because this study also observed an increase in the 4-VP-releasable form content by higher free aldehyde concentrations.

Baert et al. showed a possible aldehyde release of cysteine and bisulfite aldehyde adducts by 4-VP in beer [48]. The bisulfite adducts could be neglected in the wort samples because they foremost appear during fermentation [2]. However, we could only detect cysteinylated adducts for 3MB, PA, and methional. The formation of the adducts varied for different aldehydes, which indicated various stabilities. There was no consistent trend for different malt modification levels at the end of wort boiling. The percentage of the cysteinylated equivalents of aldehydes within their 4-VP-releasable form varied between different aldehydes. Only 1% for 3MB, 10% for methional, and 7.5% for PA were covered on average by the cysteinylated form. Therefore, presumably, the imine formation was advantaged. According to Bustillo Trueba, the study observed the low importance of cysteinylated aldehydes for the aging potential originating from wort [49].

### 3.4. Influence of Varied Reactants during Wort Boiling on Aging Potential

To verify the observed dynamic formation of the aging potential (precursors, free, and bound-state aldehydes) during the wort boiling model, boiling experiments were performed. Regarding the aging potential, this study focused on dicarbonyls as representative precursors and free aldehydes as aging indicators in the model-boiling trials. Furthermore, the observed accelerating effect of the proteolytic malt modification level on the aging potential in the final wort was simulated by spiking single substances (precursors and reactants) at the beginning of wort boiling. Thus, the hypothesis could be verified that the proteolytic malt modification level increased the disposable content of reactants and precursors at the beginning of wort boiling, resulting in an increased aging potential at the end of boiling.

**Figure 5:**
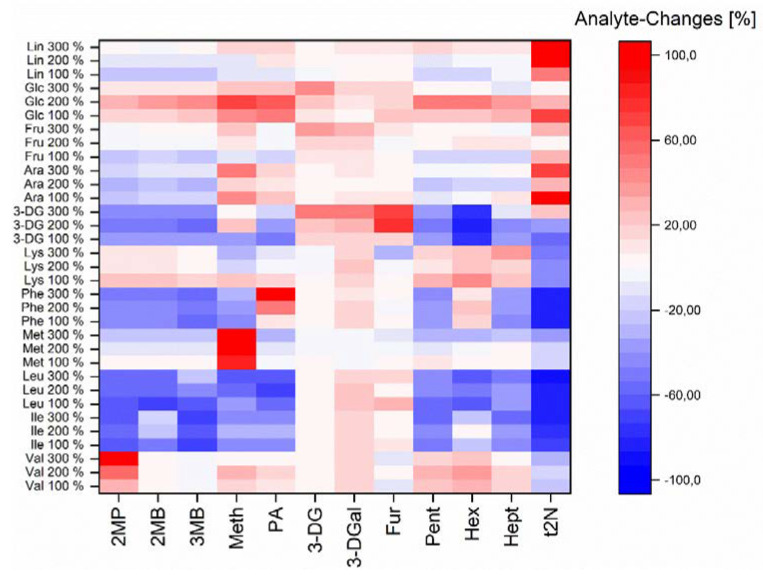
Relative changes [%] in dicarbonyl and aldehyde concentrations by artificially spiked various precursor substances after model wort boiling.

Figure 5 shows the relative changes in the aging aldehydes and dicarbonyls as precursors by spiked reactant and precursor compounds. Four groups were added to the wort on a laboratory scale. First, the amino acids (precursor and reactants): valine (Val), leucine (Leu), isoleucine (Ille), phenylalanine (Phe), and methionine (Met) were spiked to verify the Strecker degradation and lysine to verify the Maillard reaction. Second, the dicarbonyl 3-DG (precursor) was spiked to verify the Strecker degradation. The third group were the spiked sugars (reactants): arabinose (Ara), fructose (Fru), and glucose (Glc) as reactants for the Maillard reaction, and the fourth substance was linoleic acid (precursor) to verify lipid oxidation. The addition groups showed different effects on the dicarbonyls as precursors and free aging aldehydes.

Regarding the formed Strecker aldehydes, it could be shown that their corresponding amino acid (2MP: Val, 2MB: Ile, 3MB: Leu, methional: Met, and PA: Phe) forced a relative increase in the aldehyde within the spiking array (100% < 200% < 300%). That effect confirmed the Strecker degradation of the amino acids during wort boiling from their corresponding amino acid. The noticeable second effect was an absolute decrease in Strecker aldehydes by spiking Phe (despite PA), Ile, Leu, and Met (despite methional). The observed phenomena indicated the formation of imine or proline adducts, such as oxazolidinones [50], by an increased amino acid content. Therefore, the increased content of 4-VP-releasable aldehydes by higher proteolytic malt modification could be explained by enhanced adduct formation. Surprisingly, 3-DG addition decreased the Strecker aldehyde concentration. Presumably, alternative pathways, such as 5-hydroxymethylfurfrual formation or fragmentation [8], were advantaged, and the formed cleavage products possibly interacted with the Strecker aldehydes. Spiked carbohydrates slightly increased the Strecker aldehyde content. As early precursors in the Maillard reaction, they can promote Strecker degradation.

Further, the Maillard reaction products, 3-DG, 3-DGal, and furfural, were investigated. Amino acid addition showed only slightly enhancing effects on 3-DGal concentration. One explanation could be that the spiked amino acids reacted only to the Amadori compounds and not to the dicarbonyls. Further, the reaction time of 60 min did not provide enough reaction potential for forming dicarbonyls by spiked amino acids. Spiked sugars increased the dicarbonyls and furfural. Glucose and fructose are important precursor compounds and were degraded to 3-DG [14]. An increased 3-DG content was more strongly interconverted to 3-DGal.

The amino acid showed a comparable effect on the Strecker aldehydes regarding the lipid oxidation products (Pent, Hex, Hept, and t2N). Met, Phe, Ile, and Leu forced the formation of imines or oxazolidinones. The linoleic acid addition showed a strong increase in t2N and a slight increase in pentanal, hexanal, and heptanal at the Lin 300% stage. The effect confirmed the formation of these aldehydes from linoleic acid during wort boiling.

These results confirmed the hypothesis by the analytics of single precursors and aging indicators (aging aldehydes and dicarbonyls).

## 4. Conclusions

The study investigated the influence of a varied precursor concentration (amino acids and dicarbonyls) by different proteolytic malt modification levels on the formation of the aging potential during wort boiling. Summarily, it could be shown that a higher proteolytic malt modification level (calculated by soluble nitrogen content within malt specifications for brewing purposes) increased amino acid content, dicarbonyl concentration, Strecker aldehyde concentration, and the concentration of 4-VP-releasable Strecker aldehydes within malt specifications for brewing purposes. An enhancement of these substance classes lead to an increased aging potential for the final product beer out of final wort. Finally, the model-boiling studies confirmed the hypothesis that a higher amino acid content at the beginning of boiling, by an increased malt modification level, maximized the aging potential originating from the final wort toward the follow-up beer product. Some precursors or substances presented in Table 5 showed a good correlation (R^2^ > 0.8) to the soluble nitrogen content of the used malt. Table 5 shows their calculated limits in final wort according to the soluble nitrogen specifications for brewing purposes (isothermal 65 °C mashing procedure) [39,40] in this study. Other components observed in this study showed no linear dependency to soluble nitrogen content and could not be used as indicators of the aging potential due to malt modification levels. Although the calculated range was suitable for the brewing industry, the upper limit generally indicated a higher aging potential than the lower malt modification level. These values of single substances could be used as an alignment to evaluate the aging potential originating from wort for the final beer in comparable brewing setups. Further promising analytes to align the aging potential could be Amadori products, such as ε-fructoslylysine (FL), because they act as direct 3-DG precursors. They should be regarded in future studies. Thus, it could be shown that an increased proteolytic malt modification level influences the aging potential formed during wort boiling for pale lager beers.

## Figures and Tables

**Table 1:** Soluble nitrogen content of brewing malts (*n* = 3).

Variation	P1 ^1^	P2 ^1^	P3 ^1^
Target value	550 ± 25	625 ± 25	700 ± 25
B1	573 ± 10	601 ± 1	660 ± 1
B2	569 ± 3	620 ± 14	731 ± 1

^1^ Data are given in mg/100 g dry weight.

**Table 2:** Retention times (Rt) and m/z of the molecular ions of the seven cysteinylated aldehydes and caffeine.

Analyte	R_t_ [min]	[M + H]^+^
2MP-CYS	1.23	176.0740
MET-CYS	1.50	208.0460
FUR-CYS	1.61	200.0376
Caffeine	2.70	195.0877
3MB-CYS	2.94	190.0896
BEN-CYS	3.19	210.0583
PHE-CYS	3.65	224.0740
HEX-CYS	4.23	204.1053

**Table 3:** Correlation coefficients of amino acid concentration in the final wort and soluble nitrogen content.

Amino Acid	Correlation Coefficient	Amino Acid	Correlation Coefficient
Valine	0.97	Asparagine	0.64
Isoleucine	0.90	Methionine	0.52
Phenylalanine	0.87	Glutamic acid	0.46
Leucine	0.82	Proline	0.42
Threonine	0.78	Tyrosine	0.40
Lysine	0.74	Glycine	0.29
Tryptophan	0.69	Aspartic acid	0.25
Alanine	0.65	Glutamine	0.05
Arginine	0.64	Serine	0.01

**Table 4:** Formation of wort carbonyls of B1 (Avalon) and B2 (Marthe) during wort boiling, *n* = 3.

Barley Variety	Malt Modification Level	Boiling Time	2MP	2MB	3MB	Meth	PA	t2N	Pent	Hex	Hept	Fur
		min	µg/L	µg/L	µg/L	µg/L	µg/L	ng/L	ng/L	ng/L	ng/L	µg/L
B1	P1	0	535.3 ± 206.7	166.9 ± 49.7	128.6 ± 49.9	15.4 ± 3.6	64.4 ± 20.7	64.1 ± 23.2	3851.2 ± 630.5	14853.6 ± 3501.9	412.6 ± 94.1	26.7 ± 11.2
		30	59.6 ± 3.4	16.1 ± 0.6	17.8 ± 1.1	2.6 ± 0.6	10.5 ± 3.1	<LOQ	146.5 ± 14.2	709.7 ± 24.3	55.4 ± 15.8	10.3 ± 1.1
		60	120.3 ± 8.2	33.9 ± 2.3	34.0 ± 4.8	2.7 ± 0.4	11.2 ± 0.6	<LOQ	245.6 ± 32.6	1207.8 ± 202.7	88.7 ± 5.6	15.4 ± 2.2
	P2	0	496.5 ± 58.8	140.5 ± 44.5	148.2 ± 49.3	14.5 ± 3.8	46.9 ± 16.7	85.0 ± 68.7	3008.3 ± 1261.5	14429.2 ± 9114.5	230.5 ± 162.8	24.6 ± 4.3
		30	71.7 ± 15.7	15.4 ± 2.4	16.8 ± 1.1	3.9 ± 0.5	12.0 ± 0.7	<LOQ	93.8 ± 8.2	266.4 ± 19.0	14.4 ± 3.4	14.2 ± 2.8
		60	110.5 ± 6.0	26.6 ± 1.8	34.3 ± 3.8	3.0 ± 0.5	10.7 ± 1.6	<LOQ	112.8 ± 10.4	338.2 ± 74.8	20.4 ± 0.1	18.4 ± 3.3
	P3	0	995.8 ± 90.8	348.5 ± 21.7	287.8 ± 41.1	35.7 ± 6.2	108.1 ± 16.2	76.3 ± 8.5	5735.7 ± 457.5	31413.9 ± 4538.9	425.0 ± 87.7	92.22 ± 17.7
		30	91.2 ± 6.1	20.3 ± 1.0	23.9 ± 3.2	7.6 ± 1.2	22.2 ± 1.5	<LOQ	105.5 ± 4.4	283.6 ± 17.4	16.2 ± 2.6	29.2 ± 3.8
		60	155.9 ± 9.3	43.6 ± 7.0	41.8 ± 8.4	7.0 ± 1.7	20.1 ± 1.8	13.7 ± 2.0	147.3 ± 25.3	449.9 ± 83.3	21.3 ± 3.7	43.2 ± 2.2
B2	P1	0	371.9 ± 18.8	115.6 ± 5.5	112.2 ± 7.5	14.5 ± 0.4	54.7 ± 5.4	82.0 ± 10.7	3964.2 ± 441.6	23203.2 ± 2049.7	393.3 ± 28.0	32.0 ± 2.7
		30	47.2 ± 1.1	9.7 ± 0.1	8.7 ± 0.1	2.2 ± 0.4	6.9 ± 0.1	<LOQ	68.8 ± 2.1	208.0 ± 1.7	<LOQ	7.9 ± 0.3
		60	82.0 ± 3.9	17.9 ± 0.7	18.4 ± 0.4	1.9 ± 0.4	5.4 ± 0.7	<LOQ	79.8 ± 2.7	218.5 ± 11.0	<LOQ	8.4 ± 0.4
	P2	0	773.0 ± 128.4	237.3 ± 63.3	159.1 ± 28.8	38.0 ± 11.8	121.8 ± 38.0	128.7 ± 33.7	6276.1 ± 1508.0	24463.7 ± 2805.9	422.0 ± 131.5	163.7 ± 34.5
		30	137.5 ± 7.0	20.8 ± 0.6	24.6 ± 5.5	5.9 ± 1.6	15.8 ± 3.9	<LOQ	197.5 ± 26.1	476.8 ± 21.77	19.9 ± 3.7	108.5 ± 3.8
		60	191.4 ± 6.9	34.4 ± 1.4	40.3 ± 5.7	4.8 ± 0.8	13.4 ± 2.3	<LOQ	254.5 ± 2.3	340.7 ± 4.0	15.7 ± 0.6	121.5 ± 3.6
	P3	0	716.8 ± 55.1	275.8 ± 22.3	190.9 ± 22.6	49.2 ± 9.1	138.8 ± 10.9	133.8 ± 33.2	6399.0 ± 828.5	35685.8 ± 7538.9	759.0 ± 168.7	136.1 ± 30.1
		30	195.9 ± 7.7	47.5 ± 2.3	39.1 ± 1.2	15.2 ± 0.5	13.7 ± 0.4	17.0 ± 0.3	262.5 ± 8.9	306.1 ± 7.3	11.6 ± 0.8	148.9 ± 10.6
		60	233.9 ± 9.9	76.5 ± 2.9	40.7 ± 3.5	20.0 ± 1.7	61.7 ± 3.9	21.7 ± 1.2	315.4 ± 6.7	385.4 ± 19.6	14.2 ± 0.7	181.0 ± 0.9

**Table 5:** Calculated concentration limits of selected precursors and aging aldehydes in wort according to specifications of pale malts.

Substance Class	Parameter/Substance	Lower Limit	Upper Limit
Malt specifications	Soluble nitrogen [mg/100 g d.m.]	580	680
Amino acid	Valine [mg/L]	79.7	114.1
	Isoleucine [mg/L]	56.5	80.3
	Leucine [mg/L]	119.7	170.5
	Phenylalanine [mg/L]	107.2	142.3
Dicarbonyl compound	3-DG [mg/L]	12.4	20.3
Strecker aldehydes	2MP [µg/L]	112.3	192.7
	2MB [µg/L]	24.6	55.8
	Methional [µg/L]	1.7	12.3
	PA [µg/L]	6.0	37.5
	Furfural [µg/L]	20.9	116.7

## Data Availability

The data presented in this study are available within the article.

## Supplementary Materials

The following are available online at www.mdpi.com/article/10.3390/foods10102320/s1, Supplementary Excel document shows the concentrations of amino acids, free aldehydes, and 4-VP-releasable aldehydes at all sampling points during wort boiling.
